# Investigating the regulatory potential of interval training and IPA supplementation on modulating the hepatic gluconeogenesis pathway in rats with non-alcoholic fatty liver disease

**DOI:** 10.1186/s12876-026-05221-6

**Published:** 2026-08-18

**Authors:** Mohammad Hosseyn Banari, Marina Shariati, Feryal Savari

**Affiliations:** 1 Department of Physical Education and Sport Sciences, Ahv.C., Islamic Azad University, Ahvaz, Iran; 2 Department of Medical Basic Sciences, Shoushtar Faculty of Medical Sciences, Shoushtar, Iran

**Keywords:** Interval Training, IPA, NAFLD, Gluconeogenesis, G6Pase, FXR

## Abstract

**Background:**

Nonalcoholic fatty liver disease (NAFLD) is closely linked to metabolic disturbances such as hyperglycemia, insulin resistance, and obesity, yet the mechanisms underlying impaired hepatic gluconeogenesis remain unclear. This study addresses this gap by examining how interval training and indole-3-propionic acid (IPA) supplementation regulate the hepatic gluconeogenesis pathway in a rat model of NAFLD.

**Materials and methods:**

Twenty-five male Wistar rats were randomly assigned to five groups including, control, NAFLD, NAFLD + HIIT, NAFLD + IPA, NAFLD + HIIT + IPA. At the end of the experiment, lipid profile and changes FBS and insulin and HOMA-IR as well as insulin resistance were measured in serum, changes in liver weight and liver triglyceride, as well as FXR gene expression and G6Pase protein changes in the liver tissue of all rats. Oil-red O and H&E staining were used to measure the amount of fat deposition and histopathological changes in liver tissues.

**Results:**

A high-fat diet containing fructose significantly increased lipid profile, insulin sensitive parameters, as well as liver enzymes (ALP, ALT, AST) in serum, and also increased the amount of G6Pase protein and decreased FXR gene was expressed in liver tissue. However, the interaction of 8-week HIIT and IPA supplementation resulted in a significant improvement of the mentioned biomarkers, with a significant decrease in G6Pase protein levels and an increase in FXR gene expression compared to obese rats with non-alcoholic fatty liver disease.

**Conclusion:**

Based on the obtained results, it seems that the interaction of HIIT and IPA supplement consumption leads to the consumption of more circulating fuels, and at the same time, it causes a decrease in gluconeogenesis and the adjustment of blood sugar and liver status, and is probably effective in preventing the complications of obesity and non-alcoholic fatty liver.

**Supplementary Information:**

The online version contains supplementary material available at https://doi.org/10.1186/s12876-026-05221-6.

## Introduction

The incidence of non-alcoholic fatty liver disease (NAFLD) is rising worldwide, largely as a result of Western lifestyles and eating habits [1]. NAFLD, or fatty liver disease, is defined by the excessive accumulation of fat in the liver [2]. The liver modulates insulin at the peripheral level, clearing most of the secreted insulin during the first pass [3]. In NAFLD, hepatocytes undergo substantial metabolic alterations, including augmented glycolysis, gluconeogenesis, lactate production, tricarboxylic acid (TCA) cycle activity, and diminished ketone body synthesis, mitochondrial respiration, and ATP synthesis [4]. Gluconeogenesis generates glucose from non-carbohydrate substrates in the liver, maintaining glucose levels and energy balance [5]. Glucose-6 phosphate (G6Pase) is the initial intermediate in the metabolic pathway of glucose and is central to liver energy metabolism [6]. G6Pase is crucial for maintaining blood glucose levels through gluconeogenesis and glycogenolysis [7]. NAFLD is linked to insulin resistance, particularly hepatic insulin resistance (HIR), which contributes to diabetes [8]. The molecular mechanism of insulin resistance (IR) in NAFLD is intricate and encompasses many factors, including liver free fatty acid (FFA)-induced lipid toxicity, oxidative stress, endoplasmic reticulum (ER) stress, intestinal flora imbalance, inflammation, and autophagy [9]. Farnesoid X receptor (FXR) is embedded into a complex signaling network that coordinates glucose uptake, usage, and production. It is expressed in several glucose-processing organs, including the liver [10]. Hepatic gluconeogenesis generates glucose from non-carbohydrate substrates in the liver, maintaining euglycemia during fasting states. FXR, a nuclear bile acid receptor, suppresses gluconeogenesis by inhibiting key enzymes such as G6Pase, the rate-limiting terminal step that hydrolyzes glucose-6-phosphate to free glucose. FXR activation promotes small heterodimer partner (SHP) expression, which in turn represses gluconeogenic transcription factors like Forkhead box protein O1 (FOXO1) and cAMP-Response Element Binding Protein (CREB), thereby coordinating glucose homeostasis with lipid metabolism. This regulation integrates bile acid signaling with glucagon/cAMP pathways to fine-tune hepatic glucose production.

In non-alcoholic fatty liver disease (NAFLD), dysregulated FXR signaling contributes to hepatic insulin resistance and unchecked gluconeogenesis [11].

Improving HIR could treat NAFLD [9]. Lifestyle intervention is the current standard for NAFLD. The guidelines recommend weight loss through diet and exercise [12, 13]. A meta-analysis by Babu et al. [14] showed that exercise can help patients with NAFLD. Moderate-to-high-intensity exercises may be a viable alternative for managing fatty liver. High-intensity interval training (HIIT) is a time-efficient and efficacious fitness regimen [15]. The gut microbiome is the collective term for bacteria, viruses, fungi, and archaea that colonize the human gut. Microbes interact with their host in complex ways [16]. Probiotics may help restore equilibrium in NAFLD [17]. Indole-3-propionic acid (IPA) is a metabolite produced from dietary tryptophan by Clostridium sporogenes and Peptostreptococcus anaerobius. It is absorbed by intestinal cells and circulates in the bloodstream. It has anti-inflammatory properties in an animal model of steatohepatitis and reduces DNA damage and lipid peroxidation [18]. Studies in humans and rodents suggest that IPA levels may be influenced by diet. Studies suggest that IPA levels can be enhanced through dietary and lifestyle modifications. IPA levels have therapeutic potential in NAFLD [19–21].

Aberrant energy metabolism is elevated in the disease and is associated with increased hepatic steatosis and NAFLD. G6Pase is a key enzyme in hepatic gluconeogenesis. FXR regulates organelle-related pathways, including glucose metabolism. This study investigates the effect of HIIT and IPA supplementation on hepatic gluconeogenesis in rats with non-alcoholic fatty liver disease.

## Materials and methods

In the present study 25 male Wistar rats were prepared from the Ahvaz Jundishapur University of Medical Sciences, Ahvaz, Iran. Rats grouped as control (*n* = 5), NAFLD (*n* = 8), NAFLD rats plus HIIT (*n* = 5), NAFLD rats plus IPA supplement (*n* = 5) and NAFLD rats plus HIIT + IPA supplement (*n* = 5). All animals were maintained under controlled light conditions of 12/12 of light/darkness and at a temperature of 22 ± 3 °C and humidity range of 30 to 60 with free access to water and foods. High-fat/fructose diet for the obese groups and the standard diet for the normal group continued until the end of the experiment (Ethics code: IR, IAU, AHVZ.REC1403/090).

In order to prepare high-fat diet, stand flour food was purchased from Isfahan Royan Biotech Company. Then, to make 100 kg of 60% high-fat pellets, 45 kg of standard pellet powder, 30 kg of animal fat were molded into standard pellets. Moreover, to prepare a 25% solution by volume of fructose, the relationship (100x solution volume (ml)/soluble volume [13] =volume percentage) was applied to make 25% solution, 250 ml of fructose liquid dissolved in 750 ml of water. 25% fructose solution was prepared daily and provided freely in 500 ml rodent’s bottles.

Before the beginning of the training period, the running speed corresponding to maximal oxygen consumption (VO2 max) was evaluated using an incremental test. The test began with a 10 min warm-up at a speed of 5 m/min. Then, the running speed was increased by 1.8 m/min every 2 min until the animals could no longer continue running despite an increase in speed. The correlation between running velocity and VO2 max previously investigated and validated by Høydal et al. (2007), and the highest running speed achieved by the animals was recorded as the speed at VO2 max [22].

The HIIT program also included 8 weeks and 4 treadmill sessions every week. First, the rats experienced the heating process for 5 min at a speed of about 10 m per minute [22]. Then, the exercise program including 5 4-minute bouts was performed at a speed equal to 80–95% of VO2 max. Active rest periods were performed for 3 min at a speed equal to 50–60% of VO2 max. Maximal oxygen uptake (VO₂ max) was not directly measured. Exercise intensity was prescribed relative to maximum exercise capacity (MEC), determined using an incremental treadmill running test to exhaustion (6 m/min, 25% incline, with speed increases of 0.5 m/min every 2 min), a method previously validated as a reliable surrogate for VO₂ max-related exercise intensity in rats. At the end of each session, the cooling process was performed for 5 min at a speed of 5 m per minute. The duration of each HIIT session was 44 min. The slope of the machine is set to zero degrees and no changes were made to the slope of the machine until the end of 8 weeks of training [23].

### Biochemical analysis

Body weights and fasting blood sugar (FBS) levels were measured throughout the entire experimental period. Serum levels of HbA1c (Pishtazteb, Iran), insulin (Sunlong Biotech, Co, China) and lipid profile, including the total cholesterol, triglyceride (TG), low-density lipoprotein-cholesterol (LDL) and high-density lipoprotein-cholesterol (HDL) were determined by commercial kits (Pishtazteb, Iran). Fasting blood glucose level was immediately analyzed using an electronic glucometer (Diaban, SMM1000. Iran). Homeostasis model assessment of insulin resistance (HOMA-IR) was calculated by the following detailed formulas: $$\mathrm{HOMA-IR}=\text{fasting insulin}\left(\upmu\mathrm{IU}/\mathrm{dL}\right)$$$$\times\text{fasting glucose}\left(\mathrm{mg/dL}\right)/405$$.

The serum level of liver enzymes, including alanine aminotransferase [1], aspartate aminotransferase (AST) and alkaline phosphatase [24] were determined as liver injury markers using commercial kits (Pars Azmoon; IR Iran), by a serum autoanalyzer (BT- 1500-A-A, Rome, Italy) and the results are expressed as units per liter (U/l).

Oral glucose tolerance test (OGTT) was conducted during last week of the feeding period to assess the ability of the animals to dispose a glucose load. Tail vein blood samples were collected from at time 0, 30, 60, 90 and 120 min after oral administration of glucose (2 g/kg B.W.). The level of glucose in blood was measured by using commercial diagnostic kits (Pishtazteb, Iran).

### Histological evaluation

For histological analysis liver tissue slices were rapidly removed and fixed in 10% formalin. Formalin-fixed Liver tissues were exposed to various ascending alcohols (70%, 80%, 90%, and two substitutions in absolute alcohols) and subsequently infiltrated and embedded with paraffin wax. Histopathological Sect.  (5 μm thick) were obtained using a rotating microtome (MICROM model. Germany, Serial: 21074) and collected on polylysine-coated slides. The slides were oven-dried and deparaffinized with xylene and hydrated through a graded series of ethanol (100%, 95%, and 80% ethanol) and distilled water. Hematoxylin-Eosin (H&E) staining was performed according to a standard method. Blind scoring was applied under a light microscope (Olympus Optical Co. Ltd., Tokyo, Japan) to evaluate histopathological features of NAFLD assessing the percentage of total area affected by steatosis, lobular inflammation, and hepatocellular ballooning degeneration.

### Real time PCR analysis

Total RNA was extracted from frozen tissue samples with Total RNA Extraction Kit (Parstous, Iran).

The concentration and purity of RNA was measured at 260 and 280 nm wavelength by using an Eppendorf Cuvette G1.0 micro volume measuring cell (Eppendorf, Germany). The cDNA was synthesized with the use of 1 µg of RNA by using an easy™ cDNA Synthesis kit (ParsTous, Iran) and random hexamer according to the producer’s instructions. The real-time PCR analysis was performed using SYBR^®^ Green Real-Time PCR Master (ParsTous, Iran) on the StepOnePlus™ Real-Time PCR detection System (Applied Biosystems, USA). The relative expression levels of target transcripts were compared to mice GAPDH as a housekeeping gene. The primers were designed using primer3 program, ver 2.6.0 based on the sequence obtained from GeneBank (https://www.ncbi.nlm.nih.gov/gene/) (Table 1).

**Table 1:** List of mRNAs and Their Corresponding Forward and Reverse Primers

Gene name	Sequence 5’-3’	Length (bp)	Accession No.
GAPDH	F: AGTTCAACGGCACAGTCAAG	119	NC_086022.1
	R: TACTCAGCACCAGCATCACC		
FXR	F: TGAAGACCACCATCCCAGAA	142	NM_021745.1
	R: GTGCTCTGTTAGCACTCCAAA		

Frozen liver tissues were lysed using lysis buffer (Tris-HCl 50 mM, NaCl 150 mM, Triton X-100 0.1%, NaF 1 mM, supplied with protease inhibitor cocktails (Sigma-Aldrich, MO, USA)) and centrifuged at 12,000 × g for 20 min. Total protein concentrations were determined using the Bradford protein assay. Proteins were denatured, and a total of 10 µg of protein was loaded onto a 10% SDS-PAGE gel for electrophoresis. The proteins were then transferred onto polyvinylidene fluoride (PVDF) membranes (Amersham, USA) and blocked overnight at 4 °C with 5% BSA (Sigma, USA) in Tris-buffered saline (TBS, Sigma, USA) containing 0.05% Tween-20 (Sigma, USA). Membranes were incubated for 2 h at room temperature with the appropriate primary antibody anti-G6Pase (Santacruz Biotechnology Co, USA; sc-25840) diluted 1:500 in blocking solution. After three 15-min washes in Tris-buffered saline with Tween 20, membranes were incubated with a mouse anti-rabbit IgG-HRP secondary antibody diluted 1:1000 in block solution for 2 h (Santacruz Biotechnology Co, USA; sc-2357). Following three additional washes, protein reactivity was visualized using an ECL detection kit (Parstous, Iran). Protein loading was normalized to GAPDH immunoreactivity (D16H11; Cell Signaling Technology, USA). The blots were detected using the Fusion X chemiluminescence imaging system (Vilber, USA). Optical density analysis was performed using the Fusion X software and expressed as fold change the control group.

### Statistical analysis

Results were expressed as means ± standard deviation (mean ± SD). One-way analysis of variance (ANOVA), followed by tukey’s post hoc tests, was used for the identification of significant differences for multiple comparisons among different groups. All graphs and analyses were performed using GraphPad Prism version 9.0.0).

## Results

### Histopathological evaluation

**Fig. 1:**
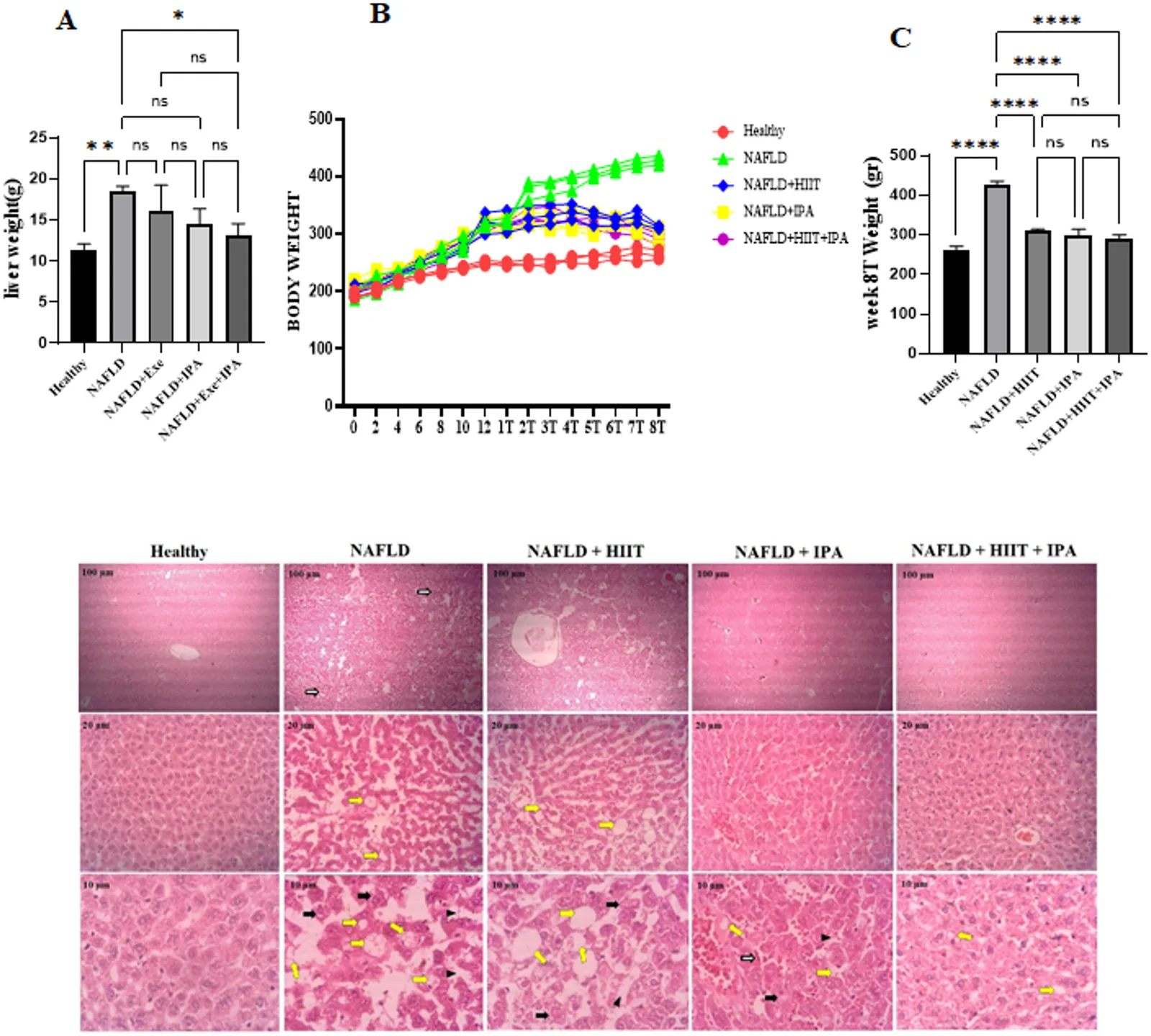
The effect of HIIT and indole propionic acid (IPA) supplementation on the histopathological features of liver in Healthy, NAFLD, NAFLD+HIIT, NAFLD + IPA and NAFLD+HIIT + IPA groups. Severe cellular swelling or balloon degeneration (black arrows), sinusoidal dilation (Arrowhead), fatty changes (yellow arrows) and mononuclear inflammatory cells (White arrows). H & E staining with a magnifications of 4×, 20×, 100 ×, and scale bars = 100 μm, 20 μm and 10 μm. The effect of HIIT and indole propionic acid (IPA) supplementation on the Liver weight (**A**), Body weight progression (**B**) and Final Body weight (**C**). ^*^P < 0.05, ^**^P < 0.01, ^***^P < 0.001 and ^****^P < 0.0001 significant differences were observed in rats that received interventions compared to rats subjected to the NAFLD model. ns: non-significant; NAFLD; non-alcoholic fatty liver disease, HIIT; high intensive interval training, IPA; indole propionic acid

The liver tissue of control group displayed normal hepatocytes and lobular architecture and surrounded by normal sinusoids (Fig. 1A). In the NAFLD group, severe cellular swelling (balloon degeneration) and sinusoidal dilation were observed versus control (Fig. 1B). In NAFLD+HIIT groups exhibited histologically moderate cell swelling (ballooning degeneration), less fatty changes, and Infiltration of mononuclear inflammatory cells (Fig. 1C). Compared to NAFLD group, supplementation with IPA reduced both steatosis and liver injuries confirmed by decreased inflammatory infiltration with mild degree of hepatocyte ballooning (Fig. 1D). Notably both ballooning degeneration and fatty change of hepatocytes substantially reduced in rats received combinatorial intervention with HIIT and IPA versus NAFLD (Fig. 1E). These findings suggest that the synergistic effects of exercise training and the immunometabolite IPA significantly ameliorate the adverse histopathological changes induced by NAFLD.

As shown in Fig. 1 (F), liver weight significantly increased in the NAFLD group as compared with the healthy group (**p < 0.01). Interventions partially attenuated this increase. Although liver weight in the NAFLD+HIIT, and NAFLD + IPA groups showed a decreasing trend compared with the NAFLD group, these differences did not reach statistical significance (ns). However, a significant difference was observed in NAFLD+HIIT + IPA group versus NAFLD (*p < 0.05).

Body weight progression throughout the experimental period is illustrated in Fig. 1 (G). All groups exhibited a gradual increase in body weight over time. The NAFLD group showed a more pronounced weight gain compared with the healthy group. Rats in the NAFLD+HIIT, NAFLD + IPA, and NAFLD+HIIT + IPA groups demonstrated moderated body weight gain relative to the NAFLD group, with the combined intervention group showing the most stable trend during the later stages of the study.

Final body weight at the end of the protocol is presented in Fig. 1 (H). The NAFLD group showed a significantly higher final body weight compared with the healthy group (****p < 0.0001). Both exercise and IPA interventions significantly reduced final body weight compared with the NAFLD group (****p < 0.0001). However, no significant difference was observed between the NAFLD+HIIT and NAFLD + IPA groups (ns). The combined intervention (NAFLD+HIIT + IPA) was significantly effecive on final body weight reduction.

### Biochemical analysis

#### Lipid profile

**Fig. 2:**
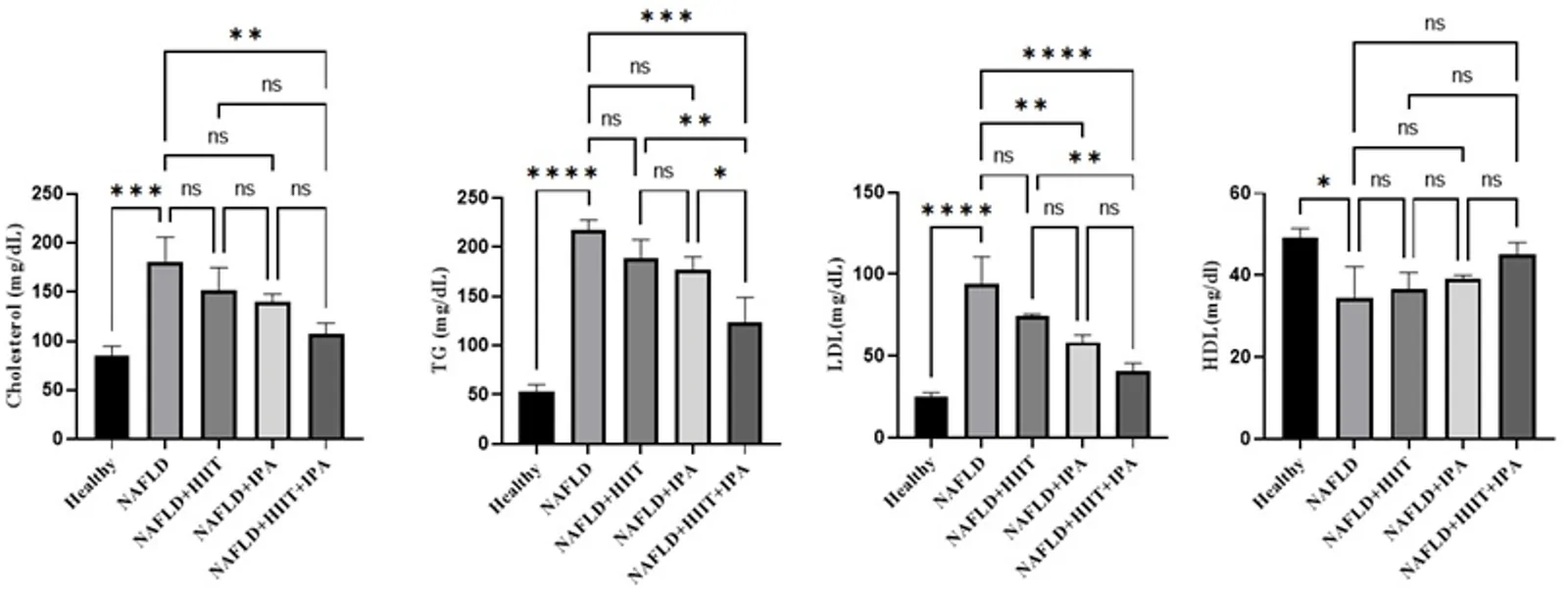
The effect of IPA and exercise training on serum concentration of TG, TC, HDL, and LDL. Data are expressed as means ± S.D, *n* = 8 in each group. ^*^P < 0.05, ^**^P < 0.01, ^***^P < 0.001 and ^****^P < 0.0001 significant differences were observed in rats that received interventions compared to rats subjected to the NAFLD model. ns: non-significant; NAFLD: Non-Alcoholic Fatty Liver Disease; IPA: 3-Indolepropionic acid; HIIT; high intensive interval training; TG: triglyceride; TC: total cholesterol; HDL: high-density lipoprotein; LDL: low-density lipoprotein

NAFLD obese rats had significant alteration in plasma lipid profile compared with the healthy group (*p* < 0.0001). In comparison to NAFLD group, serum levels of TC, TG and HDL were not significantly altered in NAFLD+HIIT group (*p* > 0.05). However, after 8 weeks of HIIT, level of LDL reduced significantly in NAFLD+HIIT group versus NAFLD (*p* < 0.05). Moreover, the result of IPA administration alone was shown to reduce TG and LDL serum levels (*p* < 0.05 and *p* < 0.01 respectively) but, TC and HDL did not significantly alter in this group as compared to NAFLD group (*p* > 0.05). As shown in Fig. 2, a comparison between a combined intervention of IPA and HIIT resulted significant improvements in these parameters versus NAFLD (*p < 0.01, ***p < 0.001). It should be noted that there were no statistically significant differences between NAFLD+HIIT + IPA and NAFLD + IPA groups for all parameters of lipid profile. However, serum lipid profile differed significantly between NAFLD+HIIT + IPA and NAFLD+HIIT groups (*p* < 0.05, *p* < 0.01).

#### Liver enzymes

**Fig. 3:**
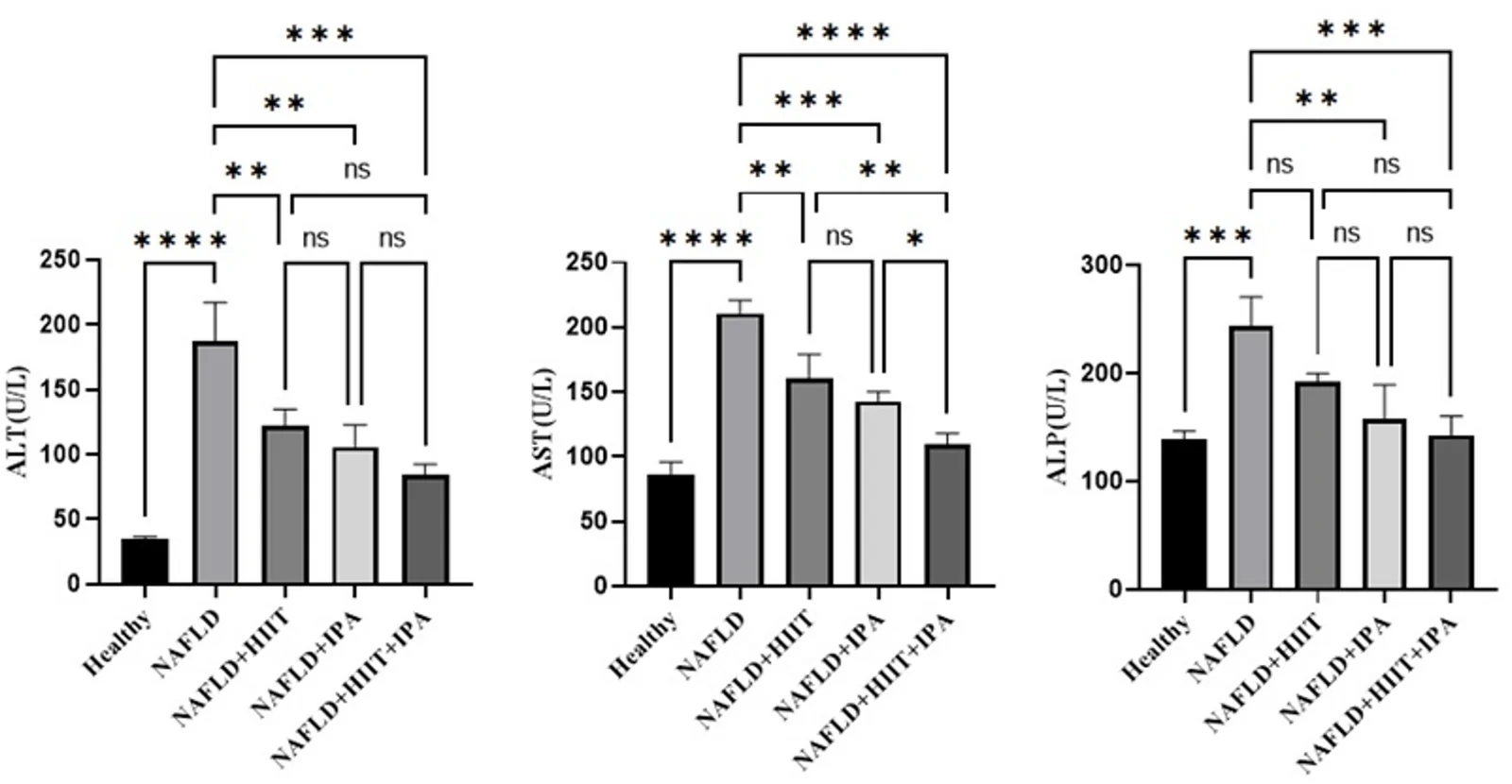
The effect of IPA and exercise training on serum level of ALT, AST, and ALP. Data are expressed as means ± S.D, *n* = 8 in each group. ^*^P < 0.05, ^**^P < 0.01, ^***^P < 0.001 and ^****^P < 0.0001 significant differences were observed in rats that received interventions compared to rats subjected to the NAFLD model. ns: non-significant; NAFLD: Non-Alcoholic Fatty Liver Disease; IPA: 3-Indolepropionic acid; HIIT; high intensive interval training; AST: Aspartate aminotransferase; ALT: Alanine aminotransferase; ALP: Alkaline phosphatase

As shown in Fig. 3 following NAFLD induction, the serum levels of ALT, AST and ALP significantly increased compared with healthy group (***p < 0.001). In comparison to NAFLD group, serum levels of hepatic enzymes significantly decreased in both NAFLD+HIIT and NAFLD + IPA groups. However, there was no statistically significant difference in this reduction between NAFLD+HIIT and NAFLD + IPA. Combined intervention with HIIT and IPA in NAFLD rats led to significant decrease in serum levels of liver enzymes group in comparison with NAFLD group. In NAFLD+HIIT + IPA, reduction of serum ALT level showed no significant difference with NAFLD + IPA and NAFLD+HIIT groups.

#### Glycemic factors

**Fig. 4:**
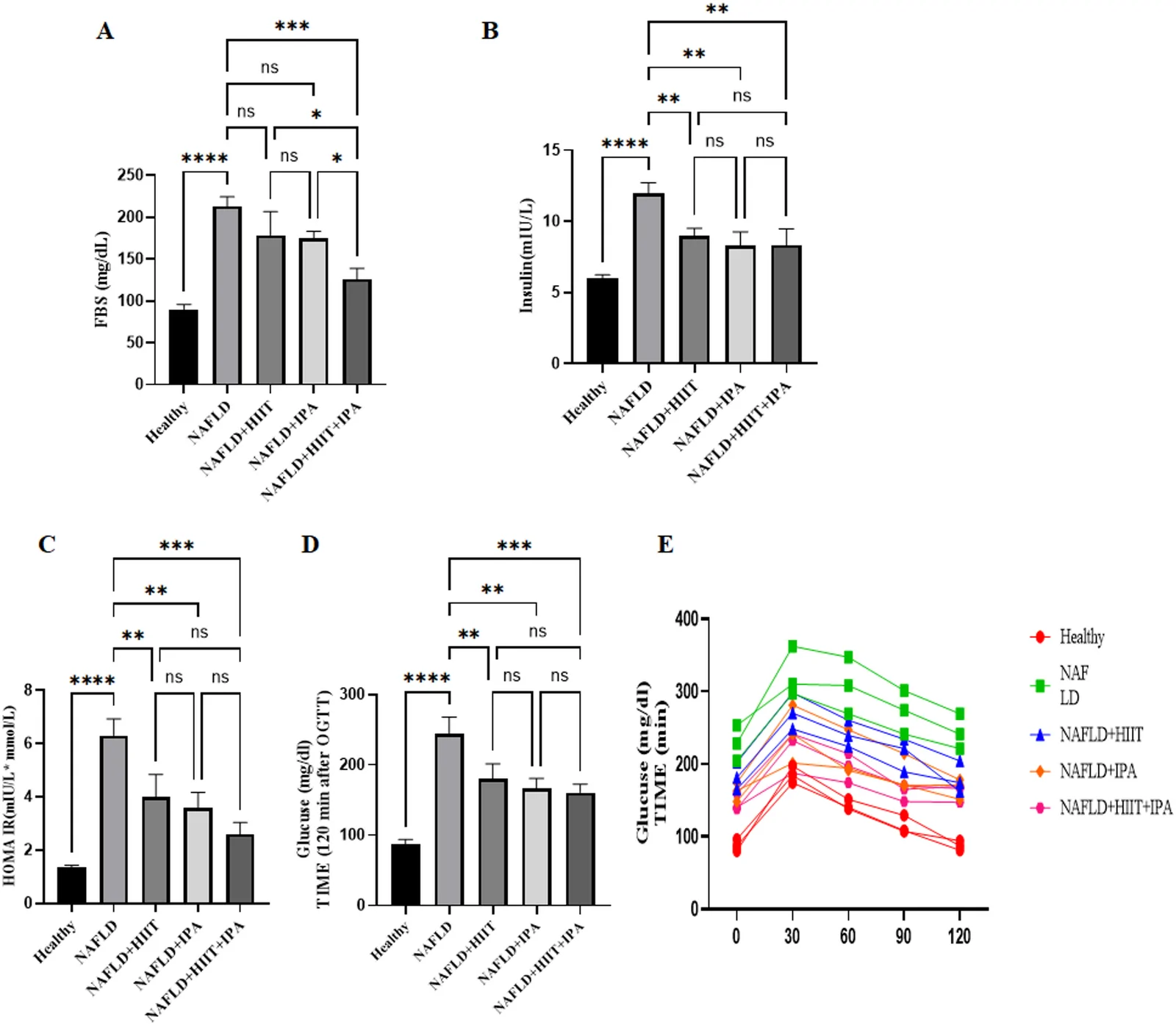
The effect of IPA and exercise training on serum level of glucose (**A**), insulin (**B**), HOMA-IR index (**C**) and OGTT (**D** and **E**). Data are expressed as means ± S.D, *n* = 8 in each group. ^*^P < 0.05, ^**^P < 0.01, ^***^P < 0.001 and ^****^P < 0.0001 significant differences were observed in rats that received interventions compared to rats subjected to the NAFLD model. ns: Non-significant; NAFLD: Non-Alcoholic Fatty Liver Disease; IPA: 3-Indolepropionic acid; Exe: Exercise; FBS: Fast blood sugar; HOMA-IR; Homeostasis model assessment of insulin resistance. OGTT; Oral glucose tolerance test; NAFLD: Non-Alcoholic Fatty Liver Disease; IPA: 3-Indolepropionic acid; HIIT; high intensive interval training

As shown in Fig. 4, the glucose level in serum of NAFLD group was higher than that in healthy group (***p < 0.001). Both NAFLD+HIIT and NAFLD + IPA groups could decrease glucose level (**p < 0.01) versus NAFLD. Compared with NAFLD group, FBS level was significantly decreased in NAFLD rats received combined intervention of HIIT and IPA (***p < 0.001). FBS level in NAFLD+HIIT + IPA was significantly lower than NAFLD + IPA (*p < 0.05). We found that, the insulin level in NAFLD group was obviously increased (Fig. 4). After intervention with HIIT, IPA supplementation and their combination, the level of insulin markedly reduced (*p < 0.05, **p < 0.01). There were no statistically differences between NAFLD+HIIT, NAFLD + IPA and NAFLD+HIIT + IPA in reduction of insulin level (*p* > 0.05).

As a result, by evaluating the serum insulin and FBS levels, we found significantly higher HOMA-IR value in rats subjected to NAFLD than that in the healthy group(****p < 0.0001). After 8 weeks of interventions, the HOMA-IR level was markedly reduced in NAFLD+HIIT + IPA group as compared with NAFLD (****p < 0.0001). HOMA-IR also decreased in NAFLD+HIIT and NAFLD + IPA groups versus NAFLD (***p < 0.001) indicating increased insulin sensitivity in treated groups. However, there was not found any significant difference between the NAFLD+HIIT and NAFLD + IPA groups.

As illustrated in Fig. 4D and E, the blood glucose concentration in healthy mice returned to baseline levels within 120 min following oral glucose administration. In contrast, mice with hepatic steatosis exhibited a significantly elevated blood glucose level at 120 min compared with healthy controls (****p < 0.0001). Notably, NAFLD+HIIT group demonstrated a significant attenuation in blood glucose levels relative to the NAFLD group (*p < 0.05). Similarly, administration of the IPA supplement in NAFLD mice resulted in a marked reduction in blood glucose concentration compared with the NAFLD group (*p < 0.05). Importantly, the combination of interval training and IPA supplementation led to a highly significant decreaseand in blood glucose levels and produced the most pronounced hypoglycemic effect, compared with the NAFLD group (***p < 0.001).

### Molecular assessments

**Fig. 5:**
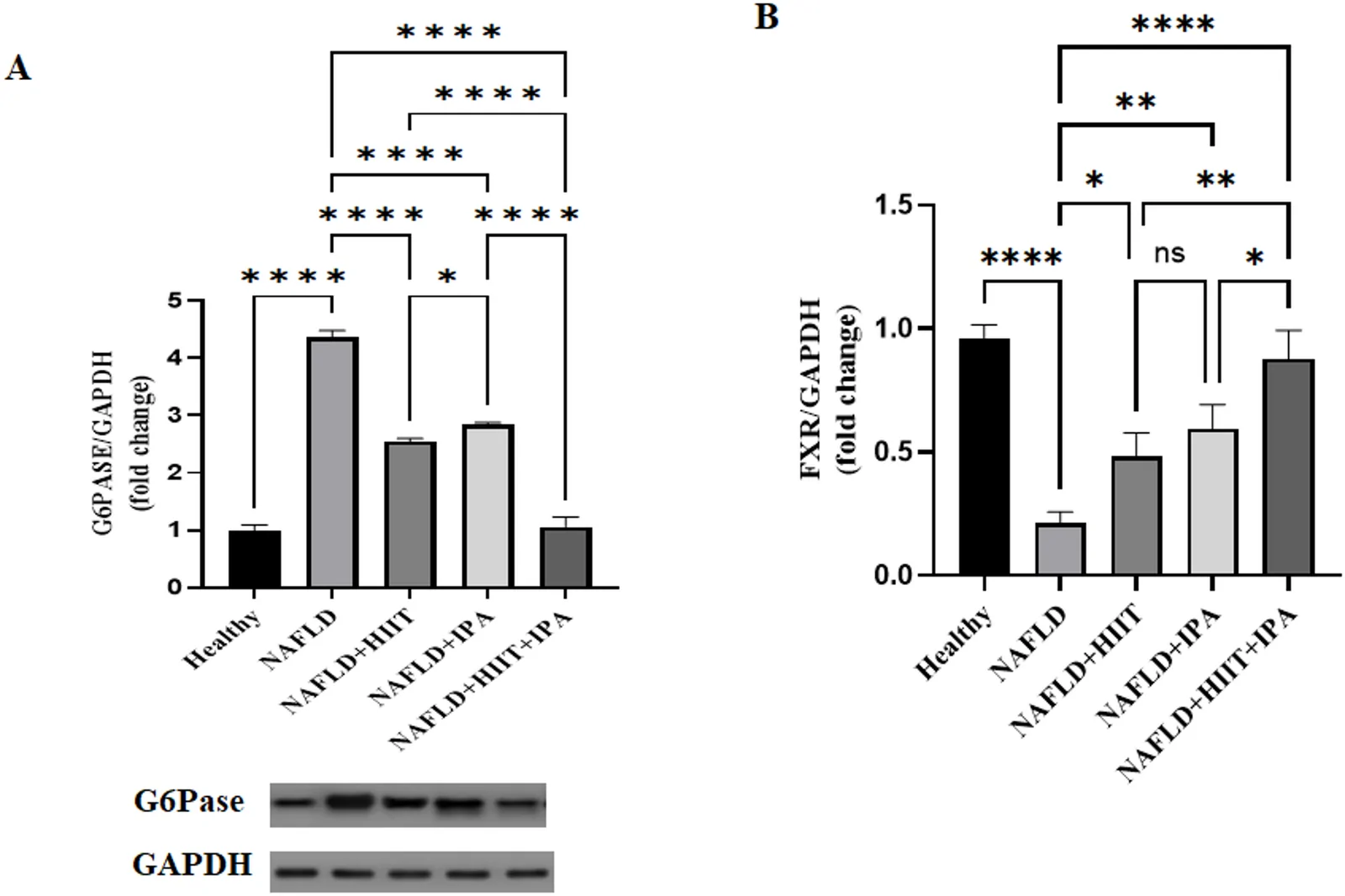
The effect of IPA and exercise training on gene expression level of FXR (**A**) and protein level of G6Pase (**B**). Data are expressed as means ± S.E.M, *n* = 8 in each group. ^*^P < 0.05, ^**^P < 0.01, ^***^P < 0.001 and ^****^P < 0.0001 significant differences were observed in rats that received IPA, exercise training and combination of IPA and exercise compared to rats subjected to the NAFLD model. ns: Non-significant; NAFLD: Non-Alcoholic Fatty Liver Disease; IPA: 3-Indolepropionic acid; HIIT; high intensive interval training; G6Pase: Glucose-6 phosphatase; FXR: Farnesoid X receptor; GAPDH: Glyceraldehyde-3-phosphate dehydrogenase

As shown in Fig. 5A and B, induction of NAFLD resulted in a significant reduction in hepatic FXR gene expression compared with the healthy control group (****p < 0.0001). Compared with the NAFLD group, FXR gene expression was significantly increased in both the NAFLD+HIIT and NAFLD + IPA groups (*p < 0.05, **p < 0.01). Although IPA supplementation showed a greater improvement in FXR expression than exercise alone, the difference between the NAFLD+HIIT and NAFLD + IPA groups did not reach statistical significance. Notably, the combined intervention (NAFLD+Exercise + IPA) led to a marked and significant upregulation of FXR expression compared with NAFLD rats (*p* < 0.001).

As illustrated in Fig. 5C and D, induction of NAFLD significantly increased hepatic G6Pase protein expression compared with the healthy group (****p<0.0001). Both exercise training and IPA supplementation significantly reduced G6Pase protein levels in NAFLD rats (****p<0.0001). Furthermore, exercise training was significantly more effective than IPA supplementation alone in lowering G6Pase expression (*p<0.05). Importantly, the combined HIIT and IPA intervention significantly reversed the NAFLD-induced elevation of G6Pase protein expression, restoring levels toward those observed in the healthy group.

## Discussion

NAFLD as a hepatic manifestation of metabolic syndrome results from disturbances in glucose and lipid homeostasis in the liver. The early stage of NAFLD defined by more than 5% of steatosis in liver parenchyma without any sing of inflammation or fibrosis [25]. Steatosis may progress to a scaring condition named NASH that can turn into fibrosis and even cirrhosis. In the last few years, NAFLD has become the world’s largest chronic liver disease [26, 27]. However, effective pharmaceutical therapies for NAFLD/NASH are not approved yet and current strategies have been proposed to target pathways directly or indirectly involved in the development of disease [28]. It seems that a combination of several beneficial therapeutic approaches can be a better and more effective approach achieve these goals compared to a single agent [29].

Gluconeogenesis as an insulin-sensitive pathway, has been suggested to be associated with NAFLD-related metabolic disturbances. Increased gluconeogenic activity, alongside elevated lipogenesis, has been reported in association with hyperglycemia, hyperlipidemia, steatosis and type 2 diabetes in the context of hepatic insulin resistance [30]. Accordingly, modulation of gluconeogenesis may represent a potential therapeutic avenue, although its precise role in NAFLD pathophysiology remains to be fully elucidated.

Modulation of gluconeogenesis by FXR activation which represses G6Pase as one of the key enzymes, provides promising therapeutic targets [31].

The importance of our work lies on the fact that it is highly likely that, IPA can serve as a substrate in the process of gluconeogenesis by altering the expression of gluconeogenic genes and improving glucose hemostasis in obese NAFLD rats as suggested by other researchers.

Under normal physiological conditions, hepatic gluconeogenesis is tightly regulated to maintain glucose homeostasis during fasting, in part through transcriptional control of key enzymes, including G6Pase, which catalyzes the terminal step of glucose production. FXR, a bile acid–activated nuclear receptor, acts as a metabolic sensor that integrates bile acid, glucose, and lipid signaling, and its activation can indirectly suppress gluconeogenesis via induction of small heterodimer partner (SHP) and inhibition of transcription factors including FOXO1 and HNF4α, thereby downregulating gluconeogenic genes such as G6Pase [10, 31].

In pathological states such as NAFLD, impaired FXR signaling has been linked to dysregulated hepatic glucose production, fasting hyperglycemia and insulin resistance, supporting a role for FXR-G6Pase crosstalk in the control of hepatic gluconeogenesis.

In our NAFLD model, we observed reduced hepatic FXR expression concurrent with increased G6Pase protein levels. This pattern is consistent with a shift in molecular markers that might reflect enhanced gluconeogenic capacity and disturbed glucose homeostasis, but these observations are correlative. Both HIIT and IPA supplementation—particularly when combined—were associated with partial restoration of hepatic FXR expression and reductions in G6Pase protein, which occurred alongside improvements in fasting glucose, insulin, HOMA‑IR, OGTT, and histological indices of steatosis. These results are consistent with the hypothesis that metabolic benefits observed after interval training and IPA are accompanied by changes in FXR‑ and G6Pase‑associated molecular pathways; however, the data do not by themselves establish direct mechanistic or causal links.

These findings suggest that the observed metabolic benefits are accompanied by FXR-associated mechanisms in which interval training and IPA synergistically reduced hepatic gluconeogenic, although direct causal relationships cannot be inferred from the present data.

Future studies employing FXR antagonists or tissue-specific FXR knockdown models are warranted to confirm the causal role of FXR in mediating these effects.

We used, for the first time, a combination of IPA supplementation and HIIT according to clinical recommendations as the effective lifestyle interventions to manage the development of NAFLD.

IPA has been associated with improvements in glucose metabolism in previous reports [32], and several studies have reported IPA‑linked reductions in hepatic steatosis and improvements in glycemic measures in diet‑induced models. Zhao et al. showed that 8-week IPA administration reversed high-fat diet (HFD)–induced steatosis, reduced hepatic lipid content, and lowered plasma liver enzymes, paralleling our observation of decreased liver triglycerides and transaminases in IPA-treated NAFLD rats [33]. Abildgaard et al. showed that IPA improved insulin resistance and glycemic variables after 6 weeks [34], which is consistent with our finding that IPA and IPA plus HIIT were associated with lower fasting glucose, insulin, and HOMA-IR. Ballanti et al. further reported that IPA improved glucose tolerance in a diet-induced obesity model, in association with modulation of Akt, a key regulator of insulin signaling [35]. These prior findings align with the direction of change observed here, where IPA (with or without HIIT) was associated with lower liver triglycerides, transaminases, fasting glucose, insulin, and HOMA‑IR. In our study these metabolic changes co‑occurred with increased hepatic FXR expression and decreased G6Pase protein; such concordance suggests a potential relationship between IPA exposure and modulation of gluconeogenesis‑related markers, but does not constitute definitive evidence of a direct regulatory axis.

Exercise interventions, including HIIT, have likewise been associated with amelioration of NAFLD‑related endpoints and with modulation of gluconeogenic enzymes [36, 37]. Prior animal and human studies report decrease in intrahepatic triglyceride content, serum ALT, visceral lipids, and insulin resistance following vigorous interval exercise. Linden et al. showed that vigorous-intensity interval exercise decreased intrahepatic triglyceride (IHTG) content and serum ALT in OLETF rats [38], which is similar to the improvements in histology and liver enzymes seen in our NAFLD+HIIT and NAFLD+HIIT + IPA groups. Abdelbasset et al. reported that HIIT reduced liver fat content, visceral lipids, insulin resistance, IHTG, and HbA1c in diabetic obese patients with NAFLD [39]. Koohestani Sini et al. found that 8 weeks of high-intensity interval exercise decreased body weight gain and HOMA-IR [24], consistent with the improved insulin sensitivity observed in our exercise-treated animals. Moreover, some experimental work has suggested exercise‑associated effects on G6Pase activity and hepatic insulin sensitivity [40, 41].

Our exercise-treated groups showed similar directional changes in metabolic and histological parameters along with FXR/G6Pase alterations; these concordant molecular and phenotypic trends are compatible with an exercise-associated modulation of pathways relevant to hepatic glucose handling, but do not definitively identify the precise molecular cascade. The combined intervention (NAFLD+HIIT + IPA) exhibited the most pronounced coordinated changes in FXR and G6Pase expression and the clearest trends toward improved steatosis and insulin sensitivity relative to monotherapies. This pattern is consistent with the hypothesis that combined lifestyle and metabolic interventions can yield additive or synergistic benefits. Nevertheless, because our observations are based on molecular and phenotypic associations, it remains appropriate to frame these effects as suggestive of a potential relationship between the interventions and FXR/G6Pase-linked metabolic pathways rather than as proof of causal regulation.

Across the study, induction of NAFLD was associated with down-regulation of hepatic FXR and concomitant increases in G6Pase protein. Conversely, interval training and IPA, especially combined, were associated with partial restoration of FXR expression and reductions in G6Pase, in parallel with improvements in glycemic indices and histology. These findings support the hypothesis that modulation of FXR signaling is associated with altered expression of gluconeogenic markers such as G6Pase and with concomitant changes in metabolic phenotype. Although plausible mechanistic intermediates include PKA- and FOXA2-sensitive gluconeogenic programs, SHP, rams, and other co-regulators that integrate bile acid and nutrient signals, our data do not directly interrogate these intermediates; therefore, such connections remain speculative and hypothesis-generating.

Based on the present results, two non-exclusive, hypothesis-driven interpretations can be proposed. First, interval training and IPA may be associated with enhanced hepatic FXR activity that coincides with reduced G6Pase expression and lower gluconeogenic output; second, improvements in insulin sensitivity and hepatic lipid status induced by these interventions may indirectly associate with the observed FXR and G6Pase changes, forming a feedback relationship. Both interpretations are consistent with the observed data but require further targeted work to delineate directionality and mechanistic detail. A limitation of the present study also is the indirect estimation of exercise intensity. It should be noted that while exercise intensity was prescribed using a well-established and validated incremental test based on maximal running speed, VO2 max was not directly measured via metabolic gas analysis. Although this indirect estimation is highly accepted in rodent models, it may limit direct comparability with studied utilizing direct calorimetry.

## Conclusion

This study examined whether IPA supplementation, alone or combined with interval training, is associated with transcriptional alterations in hepatic gluconeogenesis-related markers in a rat model of NAFLD. The interventions were associated with coordinated changes in FXR expression and G6Pase protein levels, and the combined IPA+HIIT group showed the most pronounced molecular and phenotypic trends. These findings are consistent with a potential relationship between FXR signaling, G6Pase expression, and alterations in hepatic glucose metabolism in NAFLD; however, they remain associative and hypothesis-generating.

Given that some key phenotypic endpoints did not differ markedly among intervention groups while molecular markers varied, the present data support describing G6Pase as associated with NAFLD-relevant molecular changes rather than as established causal driver of NAFLD pathogenesis. Additional work will be needed to clarify the nature and directionality of the FXR–G6Pase relationship and its relevance to NAFLD progression and therapy.

## Supplementary Information

Supplementary Material 1.

## Data Availability

All data are available from the corresponding author on reasonable request.
