# Supplementary material for: Investigating the regulatory potential of interval training and IPA supplementation on modulating the hepatic gluconeogenesis pathway in rats with non-alcoholic fatty liver disease
Source: BMC Gastroenterol. 2026 Aug 18;26:758. doi: 10.1186/s12876-026-05221-6 (PMC13761109; doi:10.1186/s12876-026-05221-6)
Supplement: Supplementary file 1 [file 12876_2026_5221_MOESM1_ESM.pdf]

Figure 5

G6Pase

M 1 2 3 4 5 6 7 8 9
10

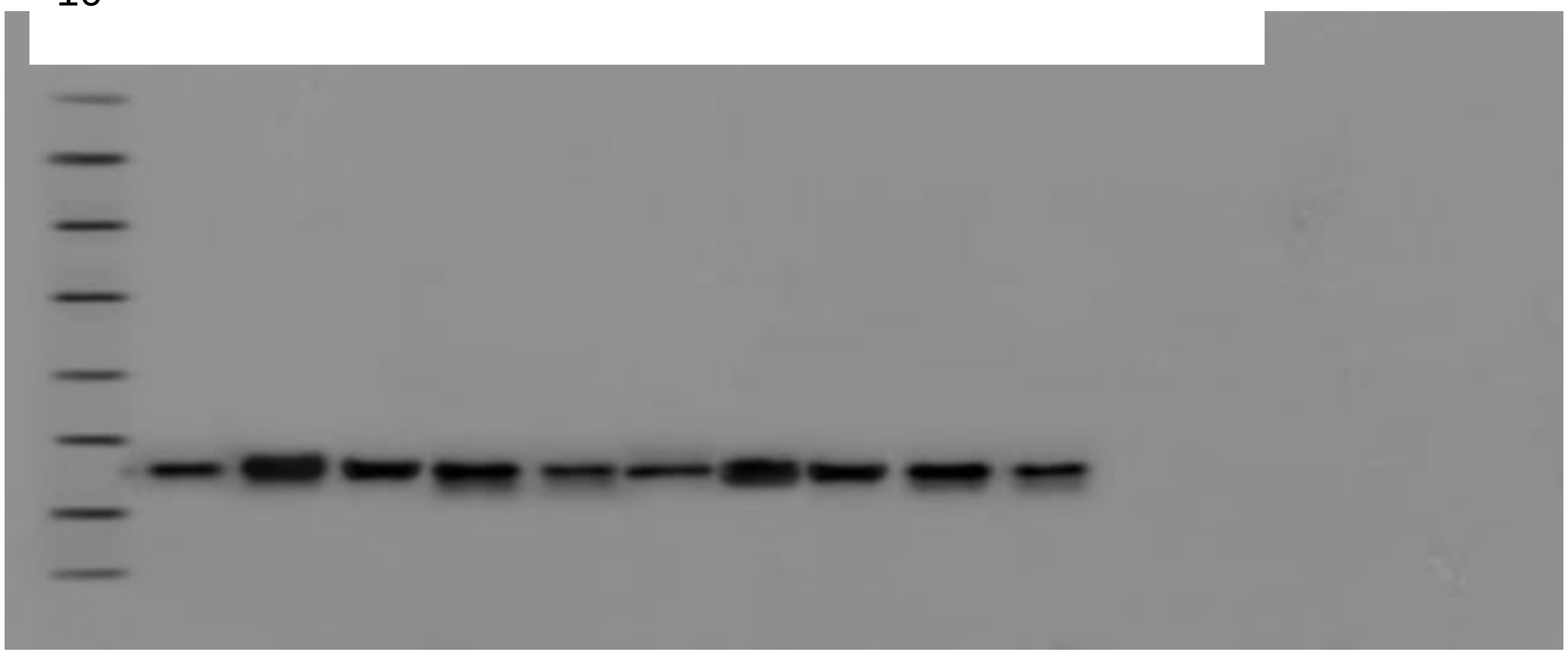

M 11 12 13 14 15

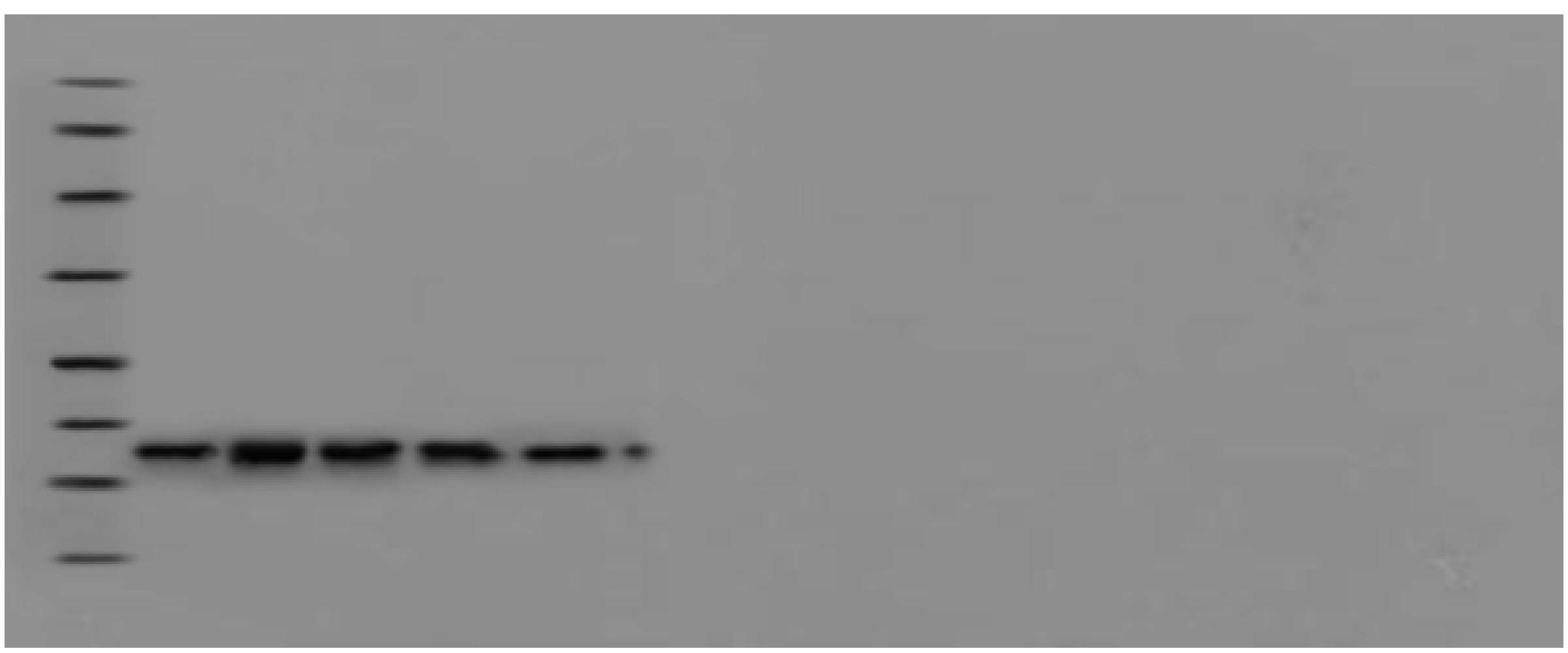

Healthy rats	1-6-11
NAFLD	2-7-12
NAFLD+EX	3-8-13
NAFLD+IPA	4-9-14
NAFLD+EX+IPA	5-10-15

GAPDH

M 1 2 3 4 5 6 7 8
9 10

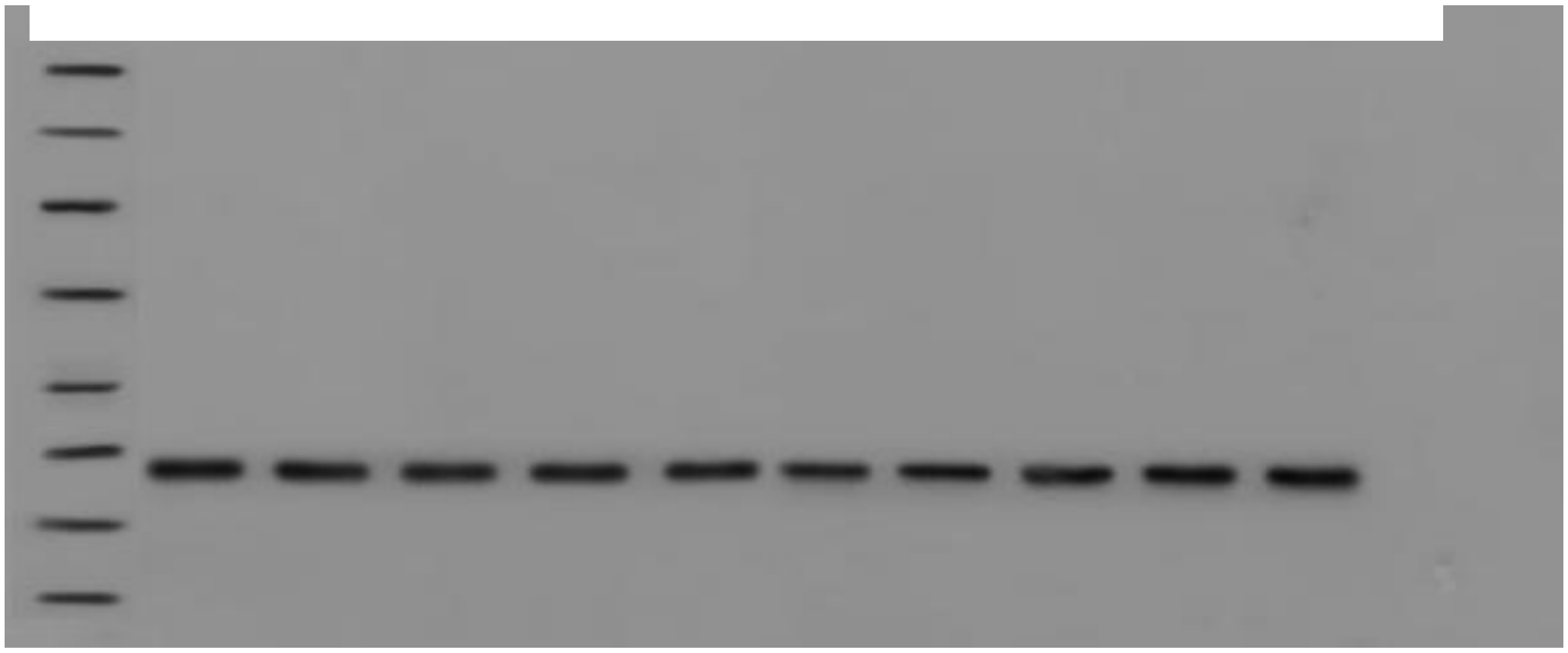

M 11 12 13 14 15

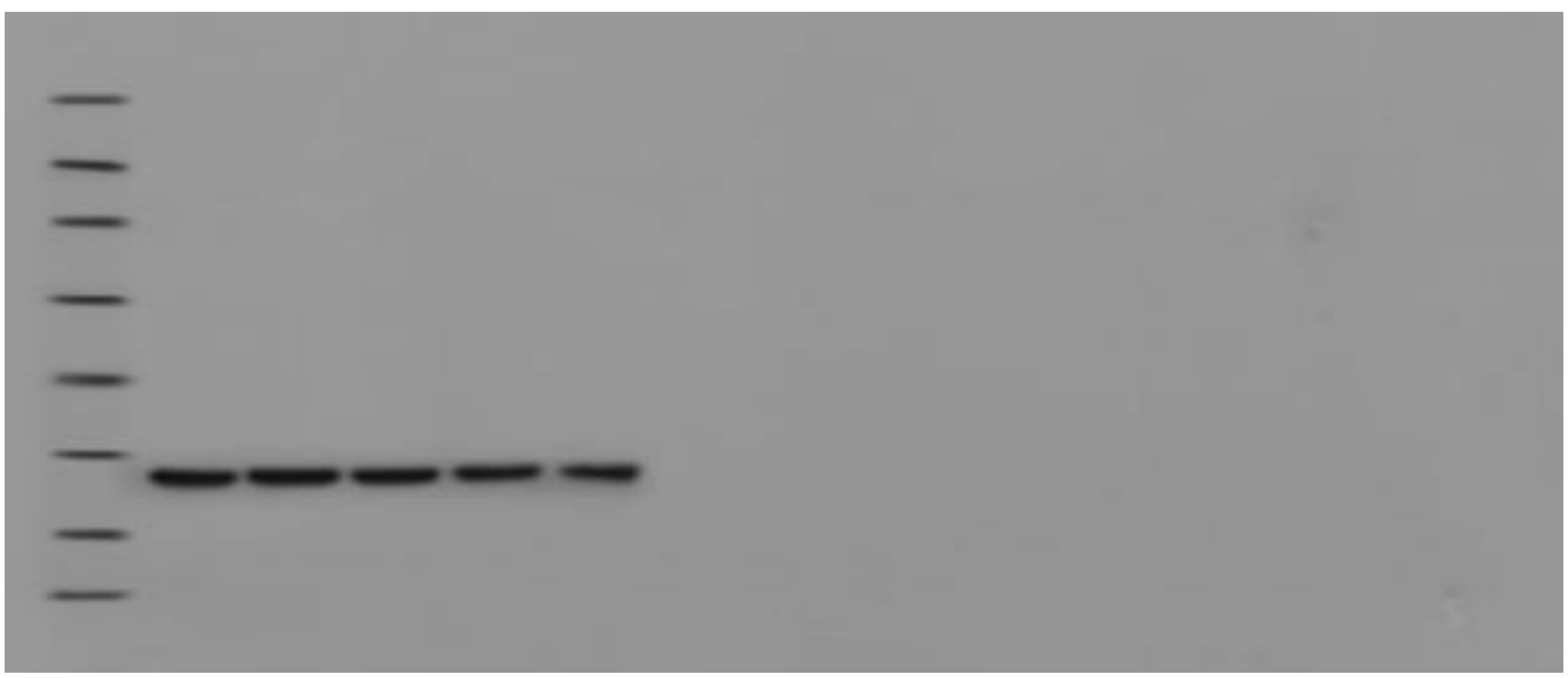

Healthy rats	1-6-11
NAFLD	2-7-12
NAFLD+EX	3-8-13
NAFLD+IPA	4-9-14
NAFLD+EX+IPA	5-10-15
